# Effect of Natural and Artificial Photoperiods and Fluctuating Temperature on Age of First Mating and Mating Frequency in the Navel Orangeworm, *Amyelois transitella*

**DOI:** 10.1673/031.011.4801

**Published:** 2011-04-13

**Authors:** Charles S. Burks, David G. Brandl, Bradley S. Higbee

**Affiliations:** ^1^ USDA-ARS, San Joaquin Valley Agricultural Sciences Center, 9611 S. Riverbend Ave., Parlier, CA 93648; ^2^ Paramount Farming Company, 33 141 E. Lerdo Highway, Bakersfield, CA 93308

**Keywords:** environmental factors, Pyralidae, seasonal effects on mating

## Abstract

The effect of weak illumination during part or all of the scotophase on mating frequency of navel orangeworm, *Amyelois transitella* (Walker) (Lepidoptera: Pyralidae), was examined in environmental chambers under long photoperiods and constant warm temperature (colony conditions) or shorter photoperiods and a cooler thermoperiod intended to mimic spring conditions in our region. These data were compared to mating frequencies in sentinel females placed in the field during the first three weeks of May. Under colony conditions weak illumination in the final hour of the scotophase resulted in ∼90% mating on the first day after eclosion; significantly greater mating compared to complete darkness throughout the scotophase, weak illumination throughout the scotophase, or weak illumination for both the first and last hour of the scotophase. In an environmental chamber programmed to simulate spring conditions, little mating occurred on the first night after eclosion and three nights were required for more than 50% of the females to mate. There was no difference in mating frequency with between moths exposed to complete darkness throughout the scotophase and those provided with weak illumination in the last half hour of the scotophase or throughout the scotophase. This delay in age of first mating was consistent with field observations with sentinel females at May in the central San Joaquin Valley. The authors conclude that, along with greater longevity and later oviposition, first mating occurs at a later age in spring conditions compared to summer conditions in this species. Planned studies of the effect of delayed mating in first and second flights will need to take these factors into account.

## Introduction

The navel orangeworm, *Amyelois transitella* (Walker) (Lepidoptera: Pyralidae), is an important pest of California almonds and several other crops (Wade 1961), and has been the target of ongoing attempts at control using mating disruption (Curtis et al. 1985; Shorey and Gerber 1996; Burks and Brandi 2004). There is evidence that, in some cases, mating disruption works by reducing and/or delaying mating rather than by preventing it; such delays in mating can profoundly impact population growth (Jones and Aihara-Sasaki 2001). Studies of effects of delayed mating on fecundity and fertility are therefore important to understanding how mating disruption works for a particular species, and laboratory studies have demonstrated such effects to varying degrees in various Lepidoptera (Fadamiro and Baker 1999; Fraser and Trimble 2001; Torres-Vila et al. 2002). These studies have typically been conducted under conditions of fixed temperature and photoperiod representative of colony conditions or the warmer portion of the growing season, but some more recent studies have examined the impact of other physiological/environmental factors such as diapause (Fitzpatrick 2006) or disease status (Evenden et al. 2005) on the effect of delayed mating on reproductive fitness.

Few such studies have examined the effect of different temperature regimes experienced by different cohorts or generations on response to delayed mating. Such studies are necessary because the question of whether to use mating disruption throughout the period in which the target pest is active (whole-season protection) or only against selected cohorts where conditions are more favorable for mating disruption (targeted protection) is an important practical consideration for mating disruption (Gut et al. 2004). In almonds, the overwintering generation of the *A. transitella* (first flight) oviposits in April and May on “mummy” nuts remaining from the previous year's crop, and the first new generation (second flight) typically begins oviposition in June (Sanderson et al. 1989). Reductions in *A. transitella* damage have been observed with mating disruption treatments beginning any time between early March and mid-May (Burks et al., unpublished data), suggesting that mating disruption might have a greater impact on this pest in the second flight compared to the first.

Seasonal variation in temperature has been shown to change diel periodicity of mating activity in the *A. transitella* (Landolt and Curtis 1982). Much research on timing of adult eclosion and mating in this species has been done in greenhouses or the laboratory. Under these conditions, adults eclose close to the beginning of the scotophase (Husseiny and Madsen 1964; Asoka Srinivasan 1970) and typically mate in the first two nights after eclosion (Wade 1961; Parra-Perazzoli and Leal 2006). Under laboratory conditions (i.e. 25–28° C constant) mating pairs generally remain *in copula* for 3 h (Parra-Perazzoli and Leal 2006). In the field, mating activity begins up to 8 h before the end of the scotophase at cooler temperatures, whereas warmer temperatures in the field or laboratory mating activity is confined to the last hour of the scotophase (Asoka Srinivasan 1970; Landolt and Curtis 1982; Parra-Perazzoli and Leal 2006). Males cease orienting to females when the temperature falls below 11 °C or with the morning twilight (Landolt and Curtis 1982).

While the age of first mating is well characterized for laboratory or warmer field conditions, few data are available concerning age of first mating under cooler conditions. The objectives of the current study were to obtain such data, to examine use of weak illumination during the scotophase to maximize mating frequency under warmer conditions, and to determine if such methods could also improve mating frequency under cooler condition.

## Materials and Methods

### Insects

Moths were maintained in a walk-in environmental chamber maintained at 26.5° C and 60% RH with a 16:8 L:D photoperiod. Intensity of illumination during the photophase ranged from 310 to 2290 lux depending on distance from overhead lights. Scotophase was below threshold (<1 lux). Larvae were reared on a wheat bran diet (11.4 1 wheat bran, 0.9 1 honey, 0.9 1 glycerin, 0.8 1 water, 100 g brewer's yeast, and 10 ml Vanderzants vitamin solution) (Tebbetts et al. 1978). Virgin moths of known sex were obtained by separating males and females as last instar larvae (Goodwin and Madsen 1964) and placing the sexes in separate 3.9 1 glass jars with approximately 2 cm of diet on the bottom to facilitate pupation. Females used in the field study came from a laboratory colony originally obtained in 1966 from the University of California, Berkeley; and those used in the laboratory study were from a strain isolated from pistachios collected from area orchards after commercial harvest and maintained in the laboratory for 1 to 2 years (9 to 18 generations) prior to the experiments.

### Mating frequency in the laboratory

A series of experiments examining mating frequency on the first night after eclosion were performed using weak illumination provided by a 4 watt, 120 volt incandescent bulb turned on and off with an electric timer.

Intensity of illumination ranged from 40 lux at 0.3 m to <1 lux at 1.8 m. The experimental unit was a 946 ml glass jar containing of 5 males and 5 females. Each morning moths eclosed the previous night (day 0) were isolated in clear 2.7 × 5 cm cylindrical plastic vials, and their sex was confirmed. Vials containing individual moths were randomized and grouped into vials containing 5 males or 5 females. Vials were then assigned in random order to a 946 ml glass canning jar with a steel mesh lids held in place by a screw top, and a cotton ball soaked in an 8% sucrose solution was placed in an inverted 2.7 × 5 cm plastic vial over the mesh top. Each jar contained a filter paper, 15 cm in diameter, folded seven times to form eight pleats, thereby providing a perch. A variable number of jars were used each day, depending on the number of moths available, and placed in the environmental chamber until the next morning with or without light from the 4 watt bulb. The first day of the experiment for each cohort was randomly assigned a treatment of either twilight or no twilight and this treatment was then alternated on subsequent days within the cohort. Mating was determined by the presence of a spermatophore in the bursa.

A series of three experiments were conducted in a walk-in chamber (2.7 m wide × 2.7 m deep × 2.8 m high inside) maintaining colony conditions (previously described) and compared some form of weak illumination during all or part of the scotophase with complete darkness during the scotophase and abrupt transition from the scotophase to photophase. For experiment 1 the alternative treatment was weak illumination beginning 1 h before photophase; this experiment was repeated for three cohorts with a total of 243 jars. For experiment 2 the alternative treatment was constant weak illumination during the scotophase; this experiment was repeated for three cohorts with a total of 230 jars. For experiment 3 the alternative treatment was weak illumination for the first hour and last hour of the scotophase; this experiment was repeated for two cohorts with a total of 173 jars. A fourth experiment was also performed in the walk-in chamber under colony conditions and directly compared mating frequency with weak illumination for both the first hour and last hour of the scotophase with frequency when weak illumination was provided for only the last hour. This experiment was examined for a two cohorts with a total of 168 jars.

A fifth experiment compared weak illumination for the last hour of the scotophase under colony conditions with the same weak illumination during the last hour of the scotophase in a reach-in environmental cabinet (0.74 m wide × 2.7 m deep × 0.70 m high inside) programmed to replicate climatically typical conditions for 15 April. Based on predicted sunrise and sunset data for Shafter, California provided by the U.S. Naval Observatory (http://www.usno.navy.mil/USNO/astronomical-applications/data-services/rs-one-day-us) and 30-year normals for high and low temperatures for Wasco, California provided by the US National Oceanic and Atmospheric Administration (cdo.ncdc.noaa.gov/cgibin/climatenormals/climatenormals.pl), this cabinet was programmed with a photoperiod of 13:11 L:D and a temperature cycle with a daily low of 7.8° for 1 h and a daily high of 25.6° C for 1; with a linear ramp from low to high temperature over the first 10 h of photophase, and a linear ramp from high to low temperature between 1 hour before the scotophase and the end of the scotophase. Jars containing 5 *A. transitella* males and 5 females were placed in the cabinet on the day of eclosion as previously described. In this smaller chamber the incandescent bulb was shield with aluminum foil to reduce illumination to <1 lux at 0.3 m. Three treatments were compared: complete darkness throughout the scotophase (24 jars), weak illumination during the final 30 min of the scotophase (24 jars), and weak illumination throughout the scotophase (31 jars).

Data were analyzed separately for each experiment using the SAS System (2008) to perform cumulative logistic regression (Agresti 2007) (GENMOD procedure) with the number of spermatophores per jars as the dependent variable and the alternative the scotophase treatments and, for experiments 1– 4, cohort as categorical predictors.

### Field study of timing and frequency of mating in spring

An experiment examining time until first mating under spring conditions was conducted using sentinel females place in the field with methods and apparatus similar to those described by Curtis and Clark (1984). A series of 16 mating stations were hung 1.5 m above the ground in 1.5 ha experimental almond breeding plot at the Parlier laboratory location. The stations were arranged in two rows of eight, with 6 m between rows and 6 to 24 m between stations within rows. On the morning of the experiment a portion of the wings on one side were clipped on freshly eclosed females, and they were then placed in mating station locations. Traps were randomly assigned to one of two treatments: a newlyeclosed female was left in the field either 1 or three nights. Four flight traps baited with virgin females as a pheromone source, as described in Burks and Brandi (2004), were also placed in the experimental almond plot. The traps were placed in two trees, one at 1.5 m and one at 3–4 m (upper canopy), and weekly counts were recorded to determine cohort phenology (i.e. flights).

Mating stations were examined each morning within an hour of sunrise, and the presence and number of *A. transitella* moths (in addition to the clipped female) was recorded. Since it wasn't always clear when moths in the field were *in copula*, this was not recorded. For traps in which the female was left in the field a single night, it was replaced on the first and second morning after the start of the experiment with another newly-eclosed virgin female. After one or three nights females were taken back to the laboratory and evaluated for mating based on the presence or absence of spermatophore(s) in the bursa. This experiment was replicated six times between 30 April and 21 May 2003. Daily low temperatures for Parlier (station 39) for this period were obtained from the California Department of Water Resources (www.cimis.water.ca.gov) and, for moths in the field for three days, the mean of the lows for the three days the moth was in the field was calculated and used.

For comparison of mating frequency, low temperatures were divided into three categories: 6 to 9° C, 9 to 12° C, and 12 to 21° C and χ^2^ analysis (PROC FREQ) was used to compare the proportion of females mated between those in the field 1 and 3 days and, for moths in the field 1 day, differences in proportion between the temperature ranges and association of mating with observation of males in the mating station in the field. Counts of males in traps were compared between weeks using a 1-way ANOVA (PROC FREQ), with counts transformed as √x (Sokal and Rohlf 1995) and the Ryan et al. adjustment was used for multiple comparisons (SAS Institute 2008).

**Figure 1.**
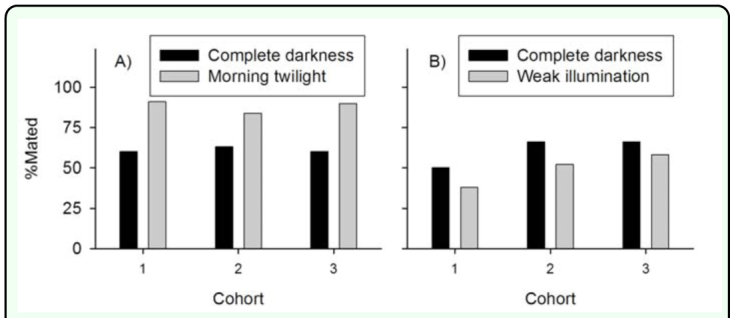
Effect of week illumination (1–40 lux) on the proportion of *Amyelois transitella* females mated I night after eclosion at 26.5° C, 16:8 L:D. (A) Complete darkness during the scotophase vs. weak illumination during the last hour of the scotophase (Twighlight AM only). (B) Complete darkness during the scotophase vs. weak illumination throughout the scotophase. In both experiments in the proportion of females mated between photoperiod treatments was significant (Cumulative logistic regression, P < 0.001). High quality figures are available online.

**Figure 2.**
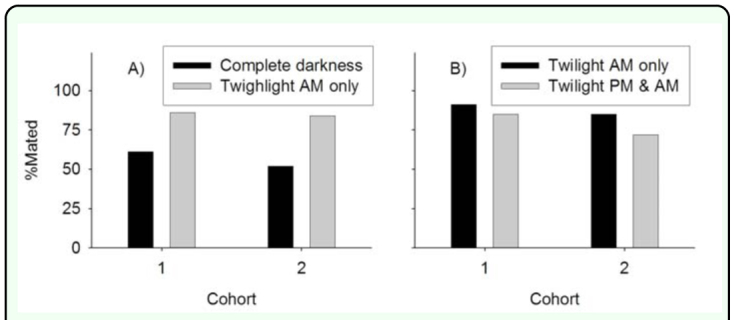
Effect of week illumination (1–40 lux) on the proportion of *Amyelois transitella* females mated I night after eclosion at 26.5° C, 16:8 L:D. (A) Complete darkness during the scotophase vs. weak illumination during the first and last hour of the scotophase vs. (diagonal lines). (B) Weak illumination during only the last hour of the scotophase (Twilight AM only) vs. week illumination during the first and last hour of the scotophase (Twilight PM & AM). In both experiments in the proportion of females mated between photoperiod treatments was significant (Cumulative logistic regression, P < 0.001). High quality figures are available online.

**Figure 3.**
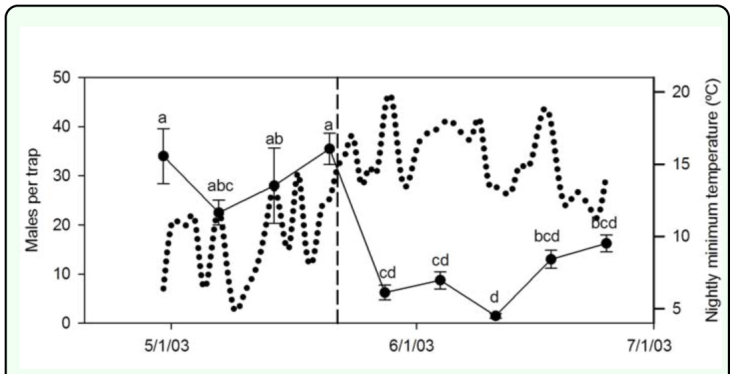
Male *Amyelois transitella* (black dots, mean ± SE) captured in flight traps baited with virgin females in the almond plot where a field experiment on mating frequency was conducted; and nightly minimum temperature (dotted line). Means not sharing the same letter are significantly different (ANOVA, *P* < 0.05). The mean males per trap did not differ significantly in the initial 3 week period in which the field experiment was conducted. High quality figures are available online.

## Results

### Mating frequency in the laboratory

Under the conditions of experiments 1 to 4, 0.10% of females were mated twice. For experiment 1, 89 ± 1.4% (mean ± SE) of females were mated with weak illumination during the last hour of the scotophase compared to 61 ± 1.4% mated with complete darkness throughout the scotophase. This difference was significant (χ^2^ = 117.85, df = 1, *P* < 0.0001), whereas the difference in mating frequency between cohorts was not significant (χ^2^ = 0.08, df =2, *P* = 0.9621) (Figure 1A). For experiment 2, 52 ± 2.1% females were mated under constant weak illumination during the scotophase compared to 59 ± 1.9% of females mated with complete darkness throughout the scotophase. This difference was significant (χ^2^ = 12.08, df = 1, *P* = 0.0005), and there was also a significant difference in the proportion of females mated between cohorts (χ^2^ = 21.48, df = 2, *P* < 0.0001) (Figure 1B). For experiment 3, 85 ± 2.3% of females were mated with weak illumination during both the first and last hour of the scotophase compared to 56 ± 2.3% mated with complete darkness throughout the scotophase. This difference was significant (χ^2^ = 85.93, df = 1, *P* < 0.0001), and there was a marginal difference in proportion of females mated between cohorts (χ^2^ = 3.41, df = 1, *P* = 0.0647) (Figure 2A). For experiment 4, 79 ± 1.9% of females were mated with weak illumination in both the first and last hour of the scotophase compared to 88 ± 1.6% mated with weak illumination in only the last hour of the scotophase. This difference was significant (χ^2^ = 12.44, df = 1, *P* = 0.0004), and there was a significant difference in the proportion of females mated between cohorts (χ^2^ = 14.70, df = 1, *P* = 0.0001) (Figure 2B). For experiment 5, an examination of effects of weak illumination in simulated flight 1 conditions showed that mating frequency was not significantly different between the three illumination treatments 1 d post eclosion (χ^2^ = 3.16, df = 1, *P* = 0.0755) and 3 d post eclosion (χ^2^ = 0.09, df = 1, *P* = 0.7587). There were significant differences between the illumination treatments for moths 2 d post eclosion (χ^2^ = 17.64, df = 1, *P* < 0.0001); females exposed to constant darkness during the scotophase did not have a significantly different mating frequency compared to those exposed to weak illumination in the last half hour of the scotophase (χ^2^ = 0.56, df = 1, *P* = 0.4537), whereas the latter treatment resulted in significantly greater mating frequency compared to moths exposed to constant weak illumination during the scotophase (χ^2^ = 11.25, df= 1, *P* = 0.0008) (Table 1).

### Field study of timing and frequency of mating in spring

The number of males captured in virgin-baited traps did not differ significantly between 30 April and 21 May (Figure 3). This observation suggests that male abundance did not differ significantly in the three-week period in which the field experiment was conducted, although factors other than abundance can affect the number of males captured in traps baited with virgin females (McNeil 1991). Of all moths recovered from field mating assays, significantly more were mated after remaining in the field three nights compared to those brought back and dissected after a single night (χ^2^ = 3.85, df = 1; *P* = 0.0496) (Table 2). There was a significant association between temperature and the proportion of females mated in the field after one night (χ^2^ = 15.92, df = 2; *P* = 0.0003), but not after three nights (χ^2^ = 1.52, df = 2; *P* = 0.4686). Within the temperature ranges, significantly more females were mated after 1 night in the field at 9 to 12° (χ^2^ = 7.19, df = 2; *P* = 0.4686), but not at 6 to 9 or 12 to 20° C.

**Table 1.**
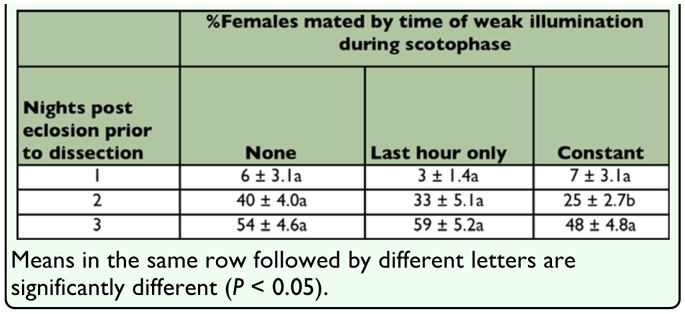
Effect of simulated spring conditions (7.8 to 25.6°C thermocycle, 13:11 L:D) in an environmental chamber on the proportion of *Amyelois transitella* mated 1 to 3 nights posteclosion.

## Discussion

Under laboratory conditions, weak illumination from an incandescent bulb during only the last hour of the scotophase significantly increased mating frequency in *A. transitella*, whereas weak illumination all night or during the first and last hour decreases it. The time of mating was not examined in this study, but a recent study examining a different field strain of *A. transitella* under very similar conditions found that 31% of mating took place between 60 and 30 min before the end of the scotophase, 57% took place in the last 30 min of the scotophase, and the remainder occurred in the 30 min after lights-on (Parra-Pedrazzoli and Leal 2006). This is consistent with previous observations by Landolt and Curtis (1982) in the field and laboratory and by Asoka Srinivasan (1970) in a greenhouse.

One possible explanation for these observations is that morning twilight is required for mating behavior when adults are held at warm constant temperatures (i.e. deprived of a thermoperiod). An earlier study of circadian effects on mating rhythm suggests the contrary; the mating rhythm was maintained for two cycles in complete darkness (Asoka Srinivasan 1970). Those observations were conducted at constant 26° C, but at a shorter photoperiod (12:12 L:D).

**Table 2.**
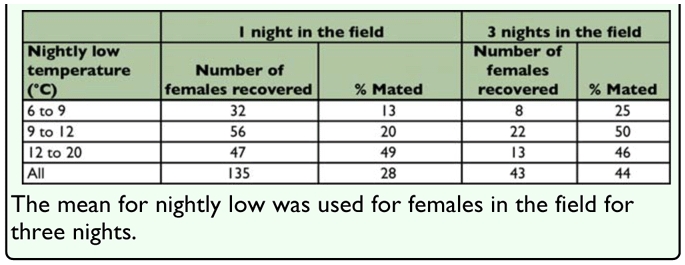
Effect of nightly low temperature and time in the field on mating frequency in sentinel female Amyelois *transitella.*

At our latitude of+36.6° N the days are 12 h or shorter between 25 September and 16 March, and in the field strain at 26.5° C diapause is induced in 100% of larvae exposed to 12:12 L:D (DGB, unpublished data). A day length of 16 h does not occur at +36.6° N. Possible explanations for the differences between the observations reported here and those in this previous study include differences in response to the endogenous circadian rhythm at the two photoperiods (Baker and Cardé 1979), and differences between laboratory strains.

Another possibility is that this species has a narrow range of light intensity required for optimum mating conditions. A recent behavioral study of courtship in *A. transitella* concluded that head-to-head contact between the male and female is an important part of mating, and that visual or tactile cues might be involved in this step (Girling and Cardé 2006). Asoka Srinivasan (1970) found optimum mating in the *P. transitella* (64%) at 0.32 lux. Mating was slightly lower at 0.17 lux (the lowest intensity tested), 44% at 6.5 lux, and 8% at 32 lux. In the cabbage looper, *Trichoplusia ni*, 0.3 lux is optimal for male response to pheromone (Shorey and Gaston 1964). While the *T. ni* does not require vision for successful mating, frequency and success of mating attempts by males is aided by shortrange vision (Shorey and Gaston 1970).

Our primary concern with determining light conditions for optimizing mating frequency was to improve mating frequency under the simulated spring conditions. While the difference between 50 and 90% mating frequency does not create insurmountable difficulties in preparing an experiment with pairs mated at a known age, the difference between 5 and 50% mating might create such difficulties. In *A. transitella*, mating activity starts earlier and occurs over a longer time in cooler conditions earlier in the season (Landolt and Curtis 1982). A similar phenomenon, in which an endogenous circadian rhythm of mating activity is modified by exogenous (environmental) factors such as temperature, day length, and light intensity has been demonstrated in many other moth species (Baker and Cardé 1979; McNeil 1991). We hypothesized that constant weak illumination might increase mating frequency in *A. transitella* under flight 1 conditions even though it had not under colony conditions. Experiment 5 showed that this was not the case. Regardless of whether the environmental cabinet was completely dark during the scotophase, weakly illuminated during the last hour of the scotophase, or weakly illuminated throughout the scotophase, mating frequency was ca. 5, 30, and 50%, respectively, for moths 1, 2, and 3 d post eclosion. However, in separate experiments using 5 males and 5 females in a jar to assess longevity and total fecundity under flight 1 conditions, 95.5% of females had one or more spermatophore(s) in the bursa when dissected at death (Burks et al., unpublished data). In that study females with access to water under flight 2 conditions lived an average of 11 d and produced half of their lifetime production of fertile eggs by the second night post eclosion, whereas females with water under colony or flight 1 conditions lived an average of 32 days and produced the first 50% of their lifetime production of fertile eggs sometime around 9 days after eclosion. These observations indicate that time until first mating, along with oviposition and longevity, were extended in simulated flight 1 conditions compared to colony or simulated flight 2 conditions.

The field observations also showed that significantly more females were mated after three compared to one night at temperatures similar to the simulated flight 1 conditions. The ranges of low temperatures used to categorize the data were selected based on the number of observations available. However, the lower two categories (6 to 9 and 9 to 12° C) happened to coincide approximately with the temperature threshold for mating activity (11° C) observed in a previous field study of mating activity in the *A. transitella* (Landolt and Curtis 1982). Simulated flight 1, with a low temperature of 7.8°, falls into the lowest of these ranges. However, in that program the temperature does not fall below 12° C until 3 h before the end of the scotophase. Since mating is presumably advanced to 8 h before the end of the scotophase under these conditions, ≥5 h are available for mating. While field conditions are more variable, it is likely that in many of the nights in the cooler ranges, temperatures initially permitted mating activity before falling below the 11° C threshold. In the field study, a feral male near the sentinel female was frequently found when checking the mating assays during the morning. One possible explanation for these observations is that, under the cooler spring conditions, males require more than one night to locate and then mate with females. However, more research is required to test this hypothesis.

In summary, we were able to use weak illumination to increase mating frequency under colony conditions, but not under simulated flight 1 conditions. Under the latter regime, mating frequency was ∼ 3% for females 1 d post eclosion and ∼ 50% for females 3 days after eclosion. Data from field experiments were similar and data from a simultaneous longevity and fecundity study not reported here indicate that most females mate before dying under the flight 1 conditions described here, and that longevity and fertility are prolonged. We conclude that studies comparing the effect of delayed mating in the *A. transitella* under flight 1 and flight 2 conditions can use 3 days after eclosion as the youngest age of mating for flight 1 conditions, whereas 1 day after eclosion is a more suitable age for this species under flight 2 conditions.
